# Immobilization of Denosumab on Titanium Affects Osteoclastogenesis of Human Peripheral Blood Monocytes

**DOI:** 10.3390/ijms20051002

**Published:** 2019-02-26

**Authors:** Felicitas Beck, Eliza S. Hartmann, Miriam I. Koehler, Julia I. Redeker, Sabine Schluessel, Baerbel Schmitt, Andreas Fottner, Marina Unger, Martijn van Griensven, Jan Michael, Burkhard Summer, Karl-Heinz Kunzelmann, Rene Beutner, Dieter Scharnweber, Paul J. Kostenuik, Susanne Mayer-Wagner

**Affiliations:** 1 Department of Orthopaedics, Physical Medicine and Rehabilitation, University Hospital, LMU Munich, Marchioninistraße 15, 81377 Munich, Germany; Huber.felicitas@googlemail.com (F.B.); elizahartmann@msn.com (E.S.H.); miriam.koehler@gwweb.de (M.I.K.); julia.redeker@icloud.com (J.I.R.); Sabine.Schluessel@med.uni-muenchen.de (S.S.); baerbel.schmitt@med.uni-muenchen.de (B.S.); andreas.fottner@med.uni-muenchen.de (A.F.); 2 Experimental Trauma Surgery, Department of Trauma Surgery, Klinikum Rechts der Isar, Technical University of Munich, Ismaninger Strasse 22, 81675 Munich, Germany; unger@uchir.me.tum.de (M.U.); martijn.vangriensven@tum.de (M.v.G.); 3 Institut für Korrosionsschutz, Dresden GmbH, Gostritzer Straße 65, 01217 Dresden, Germany; jannaj@gmx.de; 4 Department of Dermatology and Allergology, Ludwig-Maximilians-University, Frauenlobstr. 9-11, 80337 Munich, Germany; burkhard.summer@med.uni-muenchen.de; 5 Department of Conservative Dentistry and Periodontology, University Hospital, LMU Munich, Goethestraße 70, 80336 Munich, Germany; Karl-Heinz.Kunzelmann@med.uni-muenchen.de; 6 Institute of Materials Science, Max Bergmann Center of Biomaterials, TU Dresden, Budapester Straße 27, 01069 Dresden, Germany; rene.beutner@tu-dresden.de (R.B.); dieter.scharnweber@tu-dresden.de (D.S.); 7 Phylon Pharma Services, Newbury Park, CA 91320, USA; PKost@PhylonPS.com; 8 School of Dentistry, University of Michigan, Ann Arbor, MI 48109, USA

**Keywords:** nanofunctionalization, denosumab, osteoclast, titanium, implant

## Abstract

Immobilization of proteins has been examined to improve implant surfaces. In this study, titanium surfaces were modified with nanofunctionalized denosumab (cDMAB), a human monoclonal anti-RANKL IgG. Noncoding DNA oligonucleotides (ODN) served as linker molecules between titanium and DMAB. Binding and release experiments demonstrated a high binding capacity of cDMAB and continuous release. Human peripheral mononuclear blood cells (PBMCs) were cultured in the presence of RANKL/MCSF for 28 days and differentiated into osteoclasts. Adding soluble DMAB to the medium inhibited osteoclast differentiation. On nanofunctionalized titanium specimens, the osteoclast-specific TRAP5b protein was monitored and showed a significantly decreased amount on cDMAB-titanium in PBMCs + RANKL/MCSF. PBMCs on cDMAB-titanium also changed SEM cell morphology. In conclusion, the results indicate that cDMAB reduces osteoclast formation and has the potential to reduce osteoclastogenesis on titanium surfaces.

## 1. Introduction

Aseptic loosening of implants is a multifactorial process associated [1] with periprosthetic bone resorption conducted by osteoclasts [2]. The receptor activator of NF-κB ligand (RANKL) pivotally promotes osteoclast formation and activation [3] and is increased at the interface between the implant and the adjacent bone [4]. A balanced ratio of RANKL to its antagonist, osteoprotegerin (OPG), is crucial to prevent excessive osteolysis [5]. Thus, the excess of RANKL in aseptic loosening can result in focal peri-implant bone resorption [6], sometimes necessitating revision surgery. Septic loosening associated with periprosthetic joint infections has also been associated with increased RANKL and a higher RANKL:OPG ratio [7].

To mitigate this disproportional peri-implant osteoclast activity, bisphosphonates as well as recombinant OPG have been applied to implants in different studies [1,8,9,10,11,12].

In peri-implant tissues, however, there is evidence of a biological environment that does not respond to the administration of bisphosphonates with the suppression of osteoclasts [1]. Moreover, systemic bisphosphonates risk serious side effects, such as an osteonecrosis of the jaw [13] and gastrointestinal disorders. Immobilized OPG-Fc has been shown to be released from implant devices; it faces a short period of bioactivity [12] and maintains the potential of a neutralizing immune response against endogenous OPG [5], but has not successfully navigated Phase 2 clinical trials.

Utilizing the same mechanism as that of OPG, the fully human IgG monoclonal antibody denosumab (DMAB) specifically binds primate RANKL with a high degree of efficacy and has a well-established and acceptable safety profile [14]. The safety and efficacy of repeated systemic denosumab injections for periprosthetic osteolysis are currently under investigation [15], but the ability of immobilized denosumab to locally inhibit bone resorption around implants remains untested.

Modification of titanium surfaces heralded a new direction to a more favorable interaction between the implant and the periprosthetic environment. Different methods have been developed to immobilize and release bioactive molecules from titanium specimens [12,16,17,18,19,20,21,22], which facilitate a targeted therapy towards the peri-implant interface. Among different techniques, the immobilization of bioactive molecules (BAM) using oligonucleotide (ODN) strands as linker molecules constitutes a stable modification that can undergo presurgical treatment like sterilization and a subsequent fast attachment of the BAM [18]. In brief, the ODN strands are electrochemically anchored to a sandblasted, acid-etched titanium surface (SAE-Ti). Complementary ODN strands are conjugated to the BAM prior to its hybridization to the anchor strand. This nanofunctionalization could be used in order to prevent very early disproportional osteoclast activity which might develop even before osteolysis becomes evident. Our previous studies showed that in a subgroup analysis of periprosthetic tissues, the highest expression of ALP, RANK, RANKL, and TNF-α was found in the subgroup with time to revision surgery ≤2 years, whereas Cathepsin K, MMP-13, and TRAP were elevated in the subgroup with revisions ≥10 years [6].

Thus, we hypothesize that DMAB immobilized on an endoprosthetic surface could inhibit RANKL-dependent osteoclast formation and activation in the peri-implant environment at very early stages. As an oligonucleotide-mediated technique, nanofunctionalization could facilitate a targeted application of DMAB to reduce aseptic loosening with substantially lower systemic DMAB exposure, which may minimize the risk of treatment-related adverse events. As such, this study’s objective was to examine whether DMAB can be effectively nanofunctionalized on a titanium specimen in a manner that reduces osteoclast formation from stimulated human osteoclast precursor cells.

## 2. Results

### 2.1. Effect of Soluble Denosumab on Osteoclast Differentiation

Prior to testing the effect of immobilized conjugated DMAB (cDMAB), we first corroborated the ability of soluble DMAB (2 nM) to successful inhibit long-term osteoclast differentiation in vitro.

Human PBMCs were used as osteoclast precursors. The cells were cultivated in the presence of the differentiation stimulators RANKL and MCSF [23]. The long-term experiment was conducted for 28 days to analyze the effect of DMAB on the physiological period of terminal osteoclast differentiation.

**Figure 1:**
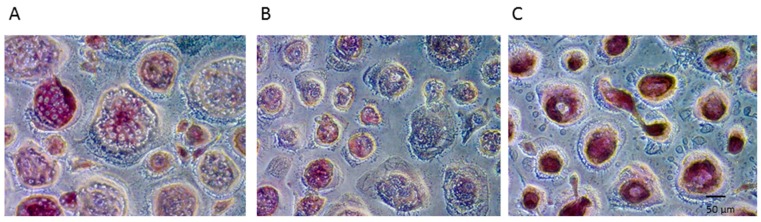
Histochemical detection of most representative staining for TRAP in PBMC -M-CSF/RANKL (**A**), PBMC +M-CSF/RANKL (**B**), and PBMC +M-CSF/RANKL + DMAB (**C**).

Histochemical detection of TRAP activity revealed a strongly positive enzymatic reaction of the stimulated PBMCs (Figure 1B). As a result, the applied osteoclast differentiation protocol was followed to generate positive control groups for the subsequent experiments. No significant enzymatic activity was detected in PBMCs exposed to the base medium only, suggesting the unstimulated precursor cells are an effective negative control for subsequent studies (Figure 1A).

In addition to the osteoclast differentiation medium, one group received 2 nM of soluble DMAB resulting in a substantially lower enzymatic reaction compared with that of the positive control (Figure 1C).

**Figure 2:**
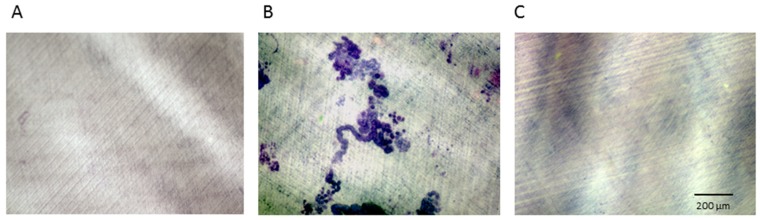
Resorptive activity of PBMC -M-CSF/RANKL (**A**), PBMC +M-CSF/RANKL (**B**), and PBMC +M-CSF/RANKL + DMAB (**C**) on dentine chips.

The depicted groups were further examined for resorptive activity on dentine chips. After 28 days, resorption pits were visualized by staining with toluidine blue. The +RANKL/MCSF-stimulated PBMCs formed Howship′s lacunae on several sites of the chips with degraded dentine appearing darkly stained (Figure 2B). The -RANKL/MCSF negative control group (Figure 2A) and the group treated with DMAB (Figure 2C) showed no signs of resorption pits.

Thus, the examination of TRAP (*n* = 6 donors) and resorptive activity (*n* = 3 donors) confirmed the eligibility of the depicted control groups for all donors. Moreover, soluble DMAB substantially reduced the accumulation of TRAP stain (Figure 1C) and prevented the initiation of resorption pits (Figure 2C) in all donors.

### 2.2. Nanofunctionalization

Prior to transferring the long-term model of terminal osteoclastogenesis to the nanofunctionalized titanium, a quantitative detection of IgG was performed to determine the amount of successfully immobilized cDMAB. DMAB was conjugated to ODN strands (cDMAB) and further applied at a concentration of 550 nM to the titanium with the complementary ODN anchor strands. The release of cDMAB was monitored after a standard curve for IgG/DMAB had been established. After the rinsing steps, 83% of the IgG corresponding to cDMAB remained hybridized to the ODN anchor strands. This was followed by an initially pronounced (70% of bound cDMA within 24 h) and then low continuous release, which was observed within 18 days.

#### 2.2.1. TRAP5b Activity

**Figure 3:**
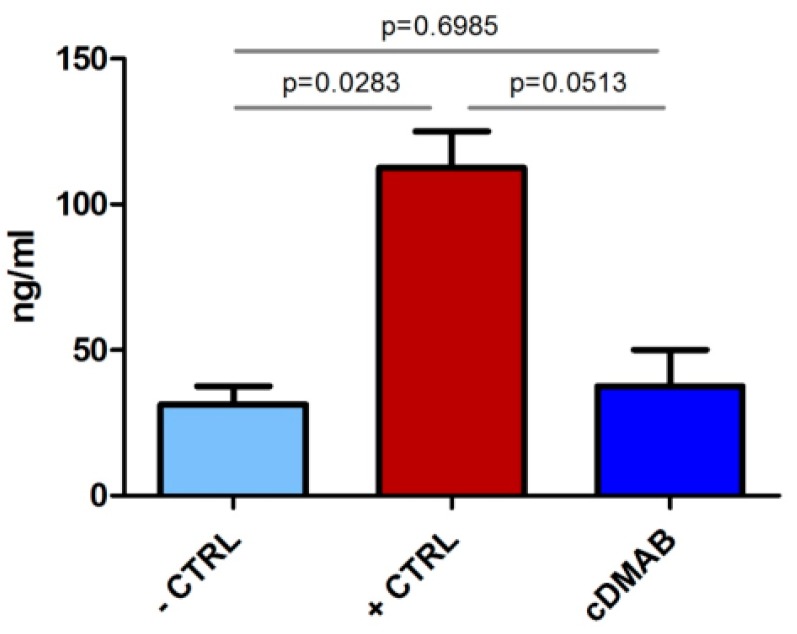
TRAP5b activity in PBMC -M-CSF/RANKL (-CTRL), PBMC +M-CSF/RANKL (+CTRL), and PBMC +M-CSF/RANKL + DMAB (cDMAB), all cultured on titanium.

Evaluation of the osteoclast-specific TRAP5b activity revealed a decreased protein level (P = 0.0513) in the cDMAB group compared to the positive control +CTRL on titanium, and almost reached the level of the –CTRL group on titanium (Figure 3).

#### 2.2.2. Endogenous Phosphatase Activity

**Figure 4:**
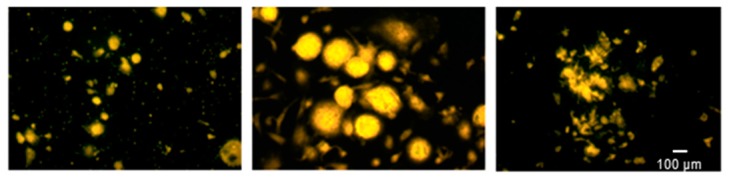
Endogenous phosphatase activity in PBMC -M-CSF/RANKL (**A**), PBMC +M-CSF/RANKL (**B**), and PBMC +M-CSF/RANKL + DMAB (cDMAB) (**C**), all on titanium.

An enzyme-linked fluorescence assay of total phosphatase activity showed that PBMCs of the +CTRL group on titanium formed large multinuclear cells (Figure 4B). PBMCs from the cDMAB group slightly clustered and presented with a reduced enzymatic reaction (Figure 4C) comparable to PBMCs from the -CTRL group (Figure 4A).

#### 2.2.3. Effect of Immobilized cDMAB on Osteoclast Morphology

**Figure 5:**
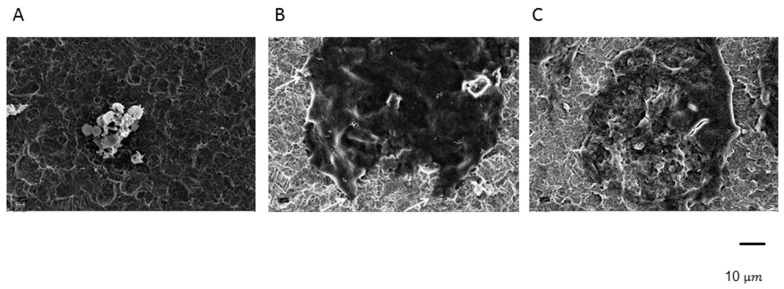
Scanning electron microscopy of PBMC -M-CSF/RANKL (**A**), PBMC +M-CSF/RANKL (**B**), and PBMC +M-CSF/RANKL + DMAB (cDMAB) (**C**), all on titanium.

Scanning electron microscopy demonstrated that PBMCs after 28 days of culture on titanium had differentiated into giant well-spread cells (Figure 5B), showing podosomes (arrows). In contrast, the -CTRL PBMCs attached to each other and formed clusters with no signs of cell fusions (Figure 5A). A change of morphology was observed in +CTRL PBMCs on cDMAB, which showed cell growth, but a notable irregularity of cell borders and surface disruptions (Figure 5C). Compared to +CTRL PBMCs, cDMAB-treated cultures exhibited a far less dense cell surface and fewer podosomes. These results suggest that osteoclast differentiation occurred in the + CTRL group. The cell size and extent of the PBMCs in the cDMAB group were similar to the +CTRL, but the PBMCs differed in morphology. These results provide a further indication that nanofunctionalized cDMAB significantly impaired terminal osteoclast differentiation.

## 3. Discussion

Enhanced bony implant fixation can be achieved by increasing new bone formation onto implant surfaces, and also by inhibiting bone resorption around implant surfaces. One therapeutic approach toward those goals is to ‘functionalize’ implant surfaces via the tethering of various bioactive molecules that can stimulate osteoblasts or inhibit osteoclasts. The present study evaluated oligonucleotide-based immobilization of the anti-RANKL antibody DMAB on a titanium surface and its effect on osteoclastogenesis from PBMCs stimulated by +RANKL/MCSF.

Oligonucleotide-based nanofunctionalization of titanium surfaces has been successfully applied to immobilize other bioactive molecules [20], for example, to increase the osteogenic activity of titanium by immobilizing bone morphogenic protein [24]. Studies on the RANKL decoy receptor OPG bound to titanium by an alkoxy silane compound [12] also show that RANKL is an effective target to locally prevent osteoclast formation and therefore potentially prevent periprosthetic osteolysis. However, OPG-Fc has not been evaluated in any late-stage clinical trials, due in part to the risk of inducing neutralizing immune responses [5], a suboptimal circulating half-life, and uncertain effects as a possible inhibitor of the TNF-related cytokine TRAIL [25]. The clinical development of OPG-based therapeutics was therefore discontinued in favor of denosumab, a fully human monoclonal antibody against RANKL that does not induce neutralizing antibodies, fails to recognize other TNF family members, and has a superior circulating half-life compared with OPG-based molecules [26]. Systemic DKAB therapy has already been contemplated as an approach to minimizing aseptic prosthesis loosening [15].

The progress of implant osseointegration and loosening is a common subject of investigation. Recent studies in a New Zealand White Rabbit model reported that several weeks are required for initial implant osseointegration [27]. The effects of DMAB [26,28] and other osteoclast inhibitors [29] on osteoclast formation have been studied for periods of less than 28 days.

The current study analyzed the effects of 28 days of DMAB exposure on the ability of cultured human osteoclast precursors to differentiate into terminally differentiated bone-resorbing osteoclasts. Soluble DMAB (2 nM) affected osteoclastogenesis and abolished the terminal differentiation of osteoclasts. Although TRAP positive multinucleated cells were found, the addition of DMAB prevented the creation of resorption pits.

Systemic DMAB therapy increases bone density and strength throughout the skeleton and also carries some risks for adverse events, which is acceptable for most patients with osteoporosis because denosumab substantially lowers the risk of fragility fractures. However, many patients receiving osseointegrating implants do not have low bone mass or increased fracture risk, and for those patients, systemic DMAB therapy would have a less favorable benefit–risk balance. The aim of this study was to establish proof of concept that DMAB can be nanofunctionally conjugated to titanium surfaces. This work represents a potentially important first step in establishing a therapeutic approach that leverages DMAB’s potent osteoclast-inhibiting effects while minimizing systemic DMAB exposure. DMAB has the potential to promote osseointegration even while it remains firmly tethered to implant surfaces by acting as a local ‘sink’ that sequesters RANKL, thereby depriving local osteoclast-lineage cells of an essential proresorptive factor. In contrast, the tethering of most other bioactive molecules, including BMPs, PTH receptor agonists, growth factors, and bisphosphonates, requires their timely release so they can directly bind to (and in some cases be internalized by) their target cells. DMAB was conjugated to ODN strands and further applied to the titanium with the complementary ODN anchor strands. It was found that DMAB remained hybridized to the ODN anchor strands and was substantially released within 18 days. To test the osteoclast-specific effect of DMAB on the differentiation of PBMCs, the tartrate-resistant acid phosphatase and its osteoclast-specific isoform 5b were used, which constitutes a hallmark of active osteoclasts [30]. As shown (Figure 3), TRAP5b protein levels were significantly decreased when M-CSF and RANKL-stimulated PBMCs were cultured on DMAB-coated titanium compared with uncoated titanium. Total phosphatase activity assessed via enzyme-linked fluorescence (Figure 4) corroborated the TRAP5b results. Collectively, the evaluation of enzymatic activity suggests that DMAB hybridized to oligonucleotide anchor strands reduces osteoclast formation by binding RANKL. Scanning electron microscopy observation after 28 days of osteoclast cultivation on DMAB-coated titanium revealed a morphology that differed from undifferentiated PBMCs as well as from osteoclast-like cells. Compared to standard PBMC cultures, cDMAB-treated cells appeared solitary, adherent to the titanium surface, and increased in size. RANKL is required for different phases of osteoclast formation and activation [3], and DMAB inhibits both differentiating and mature osteoclasts [25]. In our experiments, PBMCs were supplied with an excess of recombinant RANKL that may mimic the periprosthetic environment that has been associated with aseptic loosening [6]. Collectively with the detection of low TRAP5b in cells treated with immobilized DMAB, the morphological analysis indicates a residual effect of RANKL leading to dysfunctional osteoclasts that seem to lack terminal differentiation.

One limitation of the study was the impracticability of dentine assays after titanium cultivation, which does not rule out the possibility that some bone resorption may still occur in the presence of immobilized DMAB. However, the complete abolishment of terminal osteoclast differentiation is not the intended goal of implant functionalization, as bone turnover may contribute to bone regeneration and implant fixation [31,32]. Another possible limitation is that the majority of DMAB was released during the first few days with a small amount being continuously released during the rest of the cultivation period. Optimization strategies focusing on slower DMAB release may further enhance the inhibitory effects on osteoclast differentiation and activity. Although this in vitro study is insufficient to fully clarify inhibitory effects of immobilized cDMAB on osteoclastogenesis, it allows the hypothesis that nanofunctionalization of titanium affects osteoclastic processes. Further studies on mRNA level are necessary, as preliminary results (data not shown) could not yet prove an inhibition of osteoclast markers by cDMAB. However, mRNA data have not, to our knowledge, been used as gold standard in DMAB applications so far but osteoclast activity and protein levels.

In summary, we demonstrated that nanofunctionalized DMAB decreased the protein level of the osteoclast-specific TRAP5b and endogenous phosphatases in PBMC treated with M-CSF and RANKL for 28 days. Morphological changes in DMAB-exposed PBMCs cultured in osteoclast differentiation medium suggested an inhibitory effect of titantium-immobilized DMAB on terminal osteoclastogenesis. Collectively, these results indicate that titanium nanofunctionalized with DMAB reduces osteoclast activity by interfering with the RANKL-dependent terminal osteoclast differentiation. This in vitro study suggests that nanofunctionalization of DMAB prior to implant insertion could potentially alter the local periprosthetic milieu in a manner that favors unopposed osseointegration. Local antiresorptive treatment heralds a new direction of improving the endoprosthetic lifespan. The slow release of bioactive molecules in conjunction with improving surface osteoconductiveness may further the goals of preventing aseptic loosening.

## 4. Materials and Methods 

### 4.1. Isolation of Peripheral Blood Mononuclear Cells (PBMCs) and Osteoclast Differentiation

Human PBMCs from healthy human male donors (*n* = 8) were isolated from purchased buffy coats (Bavarian Red Cross Blood Donation Centre, Ulm, Germany; IRB approval obtained) to generate osteoclast-like cells. In brief, the leukocyte-rich blood portions were diluted in PBS (1:1) and layered on top of Biocoll Separating Solution (Biochrom, Berlin, Germany). After centrifugation at 2000 rpm for 30 min, PBMCs were isolated from the gradient and purified in several rinse steps using PBS.

To establish osteoclastogenesis, isolated PBMCs were resuspended in medium and a cell count was performed. The PBMCs were further seeded into 24-well plates as monolayer, onto dentine chips, or onto titanium specimens placed in 24-well plates at the density 6.0 × 106 cells/well. It had been shown in preliminary experiments counting the number of nonadherent cells in the supernatant after 24 h that a 2 h incubation step in the incubator at 37 °C before adding 500 μL of the medium per well was adequate to achieve sufficient adherence of the cells. All cells were supplied with alpha MEM (Biochrom, Berlin, Germany) supplemented with 10% fetal bovine serum (Biochrom, Berlin, Germany), 1% *L*-Glutamin (Biochrom, Berlin, Germany), 0.6% Penicillin/Streptomycin (Biochrom, Berlin, Germany), and 0.01% Amphotericin B (Sigma Chemical Co., St Louis, MO, USA) (base medium). The PBMCs were cultured at 37 °C and 5% CO_2_ for 28 days and the medium was replaced three times per week with additional factors. Additional factors were added to the PBMCs in four groups: (1) no RANKL, M-CSF, or DMAB (−RANKL/MCSF −DMAB: −CTRL); (2) RANKL + M-CSF, no DMAB (+RANKL/MCSF −DMAB: +CTRL); (3) RANKL + M-CSF + DMAB (DMAB); and (4) RANKL + M-CSF + conjugated DMAB (+RANKL/MCSF + conjugated DMAB: cDMAB).

The +RANKL/MCSF group was treated with an osteoclast differentiation medium, which comprised the base medium described above plus 25 ng/mL of recombinant human M-CSF (R&D Systems, Minneapolis, MN, USA), on days one and three, along with 20 ng/mL of recombinant human sRANK Ligand (PeproTech, Rocky Hill, NJ, USA) in all subsequent media changes. The RANKL/MCSF control group received the base medium only.

### 4.2. Experimental DMAB Application

To preliminarily test the effect of low-dose DMAB on osteoclast differentiation, one group of PBMCs received osteoclast differentiation medium supplemented with a single dose of 2 nM of soluble denosumab (XGEVA; Amgen, Thousand Oaks, CA, USA).

For the subsequent examination of the effect of nanofunctionalized DMAB on osteoclast formation, 550 nM of conjugated DMAB was immobilized onto titanium discs (described below); the DMAB-conjugated titanium discs of 14.7 mm diameter were cultured in osteoclast differentiation medium.

### 4.3. Osteoclast Differentiation: TRAP Histochemistry

Osteoclast differentiation was monitored by visual histochemistry-based assessment of TRAP staining (*n* = 6 donors). PBMCs were fixed and stained after 28 days of culture using a commercial kit (Sigma-Aldrich Co., St. Louis, MO), and TRAP-positivity was examined under light microscopy.

### 4.4. Osteoclast Differentiation: Dentine Resorption Lacunae

Osteoclast differentiation and activity were assessed by measuring the area of toluidine-blue-stained resorption pits on dentine chips. Ivory (from German Customs investigations) was cut into flat disks (9 × 9 × 1 mm) by a diamond-coated cutting band saw (Exakt Advanced Technologies GmbH, Norderstedt, Germany). Slices were placed into 24-well plates (Sarstedt, Nuembrecht, Germany) after disinfection (70% ethanol for 48 h).

PBMC cultures were seeded on dentin (*n* = 3 donors) in the above-mentioned groups and densities. After lysis in Tween 20 (Merck, Germany), the cells were wiped off the dentine chips with a paper towel. The chips were subsequently immersed in 1% toluidine blue (Waldeck, Münster, Germany), which revealed resorption lacunae that were quantified with the BZ9000 fluorescence microscope (Keyence, Osaka, OSK, Japan).

### 4.5. Immobilization of Oligonucleotide Strands on Titanium Specimen

**Figure 6:**
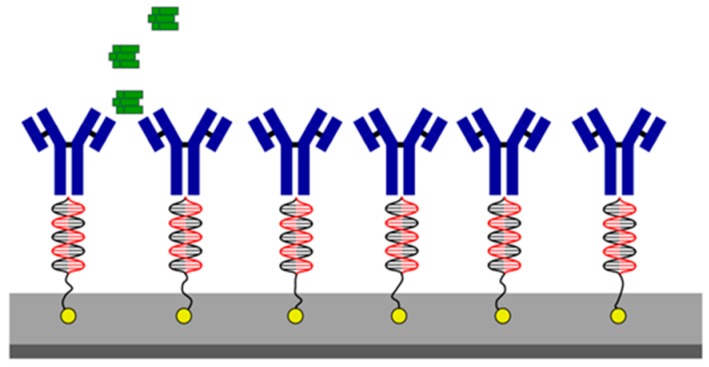
Schematic image of cDMAB immobilization onto titanium.

Titanium alloy discs (14.7 mm × 1.9 mm) were made of the alloy TiAl6V4 and ground with SiC paper P600 (26 μm) (Aesculap, Tuttlingen, Germany). The immobilization of the oligonucleotide anchor strands on the titanium specimens (Figure 6) was performed at the Max Bergmann Center for Biomaterials (TU Dresden). To immobilize the anchor strands, the sandblasted, acid-treated titanium TiAl6V4 specimens were attached to the bottom of a specially designed electrochemical cell with a three-electrode arrangement. For initial adsorption of anchor strands, the cell was filled with an electrolyte solution containing the 60mer oligonucleotide anchor strand (Thermo Fisher Scientific GmbH, Ulm, Germany). The adsorption was followed by anodic polarization to fix the anchor strands in the oxide layer of the titanium. Subsequently, the test pieces were washed and air-dried. DSS was used as a connecting molecule to conjugate DMAB to the 31mer oligonucleotide strands complementary to the anchor strands (Biomers GmbH, Ulm, Germany). The oligonucleotide strands were first activated with DSS in 1 mM acetate buffer at pH 4.0 and then mixed with denosumab (in PBS with 2 M sodium chloride, pH 7.4). This was followed by an 18 h incubation in the dark at room temperature.

### 4.6. Hybridization of the Denosumab–ODN Conjugates with Anchored Oligonucleotides

The hybridization of denosumab–oligonucleotide conjugates to the immobilized anchor strands was carried out directly before the cell colonization of the titanium specimens. The materials used were provided by the Max Bergmann Center for Biomaterials (TU Dresden). Titanium specimens were treated with 50 µL of a 550 nM denosumab conjugate hybridization solution consisting of 5 µL 10× PBS buffer (18 g sodium chloride, 1.78 g disodium hydrogen phosphate × 2 H_2_O and 0.462 g potassium dihydrogen phosphate and 200 mL deionized water of total pH 6.85), 1.1 µL conjugate solution, and 43.9 µL sterile ultrapure water. The γ-sterilized titanium specimens with the immobilized oligonucleotide anchor strands were completely wetted with the hybridization solution and incubated in the dark at room temperature for one hour. Subsequently, the samples were washed four times in PBS. The last rinse took place with sterile ultrapure water. Cells were cultured for 28 days on titanium in +RANKL/MCSF and −RANKL/MCSF groups. The conjugated titanium cells were treated with osteoclast differentiation medium (+RANKL/MCSF).

### 4.7. Quantitative Detection of DMAB

DMAB is a monoclonal antibody of the IgG2 isotype, which can be measured using an enzyme-linked immunosorbent assay (ELISA) for quantitative human IgG2 detection. Thus, the amount of DMAB rinsed from the titanium during the hybridization process was measured using an ELISA kit (eBioscience Affymetrix, Vienna, Austria) according to the manufacturer’s instructions.

### 4.8. TRAP Staining and Enzyme-Labeled Fluorescence of Phosphatase Activity

The staining of tartrate-resistant acid phosphatase [25] in monolayer cultures was performed by using a TRAP detection kit (Sigma-Aldrich Co.) to detect a change in enzymatic activity of stimulated osteoclast precursor cells when treated with DMAB.

To depict endogenous phosphatases on the titanium test specimens, the cells were fixed analogously to the TRAP staining using a citrate and acetone solution, and after 20 min of air drying, the ELF® 97 Endogenous Phosphatase Detection Kit (Molecular Probes; Eugene, OR, USA) was used according to the manufacturer’s instructions.

Results were visualized by confocal microscopy (Zeiss LSM 710).

### 4.9. TRAP5b Secretion

Secretion of the tartrate-resistant acid phosphatase isoform 5b into the cell supernatant was measured using the TRAP5b ELISA kit (BlueGene Biotech, Shanghai, China) after 28 days of culture, following the manufacturer’s instructions.

### 4.10. Scanning Electron Microscopy

After fixation in a 4% formalin solution for 24 h, the cells cultured on the titanium were dehydrated through a graded series of ethyl alcohols (50–96%) and further processed in a Critical Point Dryer. The titanium specimens were subsequently sputter-coated with gold and observed under a scanning electron microscope (Zeiss Typ LEO 1455VP).

### 4.11. Statistics 

Inferential statistical comparisons of the three groups were made using one-factorial variance analysis using the GLM procedure of the SAS 9.4 Statistical Analysis System for Windows (SAS Institute, Cary, NC, USA). All values included in these analyses were averages of at least three replicate measurements. The first global test was an F-test at the alpha level of 5%. Significant failure resulted in post hoc pairwise, two-sided group comparisons with Sidak correction. Corrected p-values below 5% were considered significant.

## 5. Conclusions

The results indicate that DMAB can be electrochemically coated onto titanium surfaces without losing its functionality. Immobilization of DMAB resulted in a reduction of terminal osteoclast differentiation from M-CSF- and RANKL-stimulated PBMCs. The inhibitory effects of DMAB appear to persist for a sufficient duration to potentially improve early implant osseointegration. The technique allows a one-time, low-dose application of DMAB, thereby minimizing systemic effects of the drug. Collectively, endoprosthetic nanofunctionalization of DMAB capitalizes on the benefits of a local therapy without compromising the efficacy of the treatment and thus has potential as a novel and clinically relevant approach to reduce the risk of aseptic loosening.

## Abbreviations

MCSF	Macrophage colony-stimulating factor
RANK-L	RANK-Ligand
TNF	a Tumor necrosis factor alpha
OPG	Osteoprotegerin
PBMCs	Peripheral mononuclear cells
a-MEM	a-Minimal Essential Medium
DMEM	Dulbecco’s Minimal Essential Medium
PBS	Phosphate-buffered saline
RT	Room temperature
TRAP	Tartrate-resistant acid phosphatase
